# The severity and status of eating disorder NOS: Implications for DSM-V

**DOI:** 10.1016/j.brat.2007.01.010

**Published:** 2007-08

**Authors:** Christopher G. Fairburn, Zafra Cooper, Kristin Bohn, Marianne E. O’Connor, Helen A. Doll, Robert L. Palmer

**Affiliations:** a Department of Psychiatry, Oxford University, Warneford Hospital, Oxford OX3 7JX, UK; b University Department of Health Sciences, Brandon Unit, Leicester General Hospital, Leicester LE5 4PW, UK

**Keywords:** Anorexia nervosa, Bulimia nervosa, Eating disorder, DSM-V, Diagnosis, Classification

## Abstract

“Eating disorder NOS” is the most common eating disorder encountered in outpatient settings yet it has been neglected. The aim of this study was to describe the characteristics of eating disorder NOS, establish its severity, and determine whether its high relative prevalence might be due to the inclusion of cases closely resembling anorexia nervosa or bulimia nervosa. One hundred and seventy consecutive patients with an eating disorder were assessed using standardised instruments. Operational DSM-IV diagnoses were made and eating disorder NOS cases were compared with bulimia nervosa cases. Diagnostic criteria were then adjusted to determine the impact on the prevalence of eating disorder NOS. Cases of eating disorder NOS comprised 60.0% of the sample. These cases closely resembled the cases of bulimia nervosa in the nature, duration and severity of their psychopathology. Few could be reclassified as cases of anorexia nervosa or bulimia nervosa. The findings indicate that eating disorder NOS is common, severe and persistent. Most cases are “mixed” in character and not subthreshold forms of anorexia nervosa or bulimia nervosa. It is proposed that in DSM-V the clinical state (or states) currently embraced by the diagnosis eating disorder NOS be reclassified as one or more specific forms of eating disorder.

## Introduction

“Not otherwise specified” (NOS) diagnoses within DSM-IV are designed for disorders of clinical severity that fall outside the specified diagnoses (American Psychiatric Association, 1994). For example, within the diagnostic classes of anxiety disorders and mood disorders are the diagnoses anxiety disorder NOS and mood disorder NOS respectively, and these exist alongside the specified individual anxiety disorder and mood disorder diagnoses. Thus, NOS diagnoses are by definition residual categories and, possibly as a result, they tend to be neglected (Pincus, Wakefield Davis, & McQueen, 1999).

An anomalous situation exists within the eating disorders in that “eating disorder NOS” is the most common eating disorder diagnosis encountered in routine clinical practice: it is considerably more common than the two specified eating disorders, anorexia nervosa and bulimia nervosa (Button, Benson, Nollett, & Palmer, 2005; Martin, Williamson, & Thaw 2000; Ricca et al., 2001; Turner & Bryant-Waugh, 2004). Despite this, there have been very few systematic descriptions of the clinical characteristics of patients with eating disorder NOS, and those that exist have had shortcomings including the use of relatively small and unrepresentative patient samples, the collection of a limited range of descriptive information, and reliance on weak or unstandardised measures. Two exceptions are of note. Ricca et al. (2001) recruited 95 first referrals with eating disorder NOS and assessed them using the Eating Disorder Examination (EDE) interview (Fairburn & Cooper, 1993). Their sample was likely to have been biased, however, as it came in part from referrals to a private clinic and as patients taking antidepressant medication were excluded. Turner and Bryant-Waugh (2004) also used the EDE to characterise cases of eating disorder NOS but their sample excluded those with binge eating disorder and those with co-existing obesity. Also the clinical description was confined to current eating disorder psychopathology.

The present study had three aims. The first was to describe the clinical characteristics of eating disorder NOS by recruiting a large and representative patient sample and assessing it using standardised measures of eating disorder-specific and general psychopathology. The second aim was to assess the severity of eating disorder NOS by comparing these cases with those with bulimia nervosa, a common eating disorder and one of established severity. The third aim was to determine whether the high relative prevalence of eating disorder NOS might be due in part to the inclusion within this diagnosis of cases closely resembling anorexia nervosa and bulimia nervosa, cases that might be better reclassified as such (Fairburn & Bohn, 2005).

## Method

### Participants

The sample comprised consecutive referrals to two eating disorder clinics in the UK, one serving central Oxfordshire and the other serving Leicester city. Both clinics provide the only secondary adult eating disorder service for the locality. Patients were included if they met the following criteria: aged 18–65 years, judged to have an eating disorder of clinical severity by one of three senior specialists in the field (ZC, CGF or RLP), and a body mass index (BMI) between 16.0 and 39.9. Each referral was evaluated by one of the three senior clinicians who established whether the patient met the inclusion criteria listed above and obtained the history of the eating disorder. Then, after the patients had been provided with a complete description of the study, written informed consent was obtained. Finally, the patients were assessed using the instruments described below. The study was approved by the two local research ethics committees.

### Assessment

The participants were assessed using the EDE interview (Fairburn & Cooper, 1993) and the following self-report measures: the Brief Symptom Inventory (BSI) (Derogatis & Spencer, 1982) which is a short version of the Symptom Checklist-90 (Derogatis, 1977), the Rosenberg Self-esteem Scale (RSE) (Rosenberg, 1965), and the UK version of the self-report Social Adjustment Scale (SAS) (Cooper, Osborn, Gath & Feggetter, 1982). The EDE was used to generate operational diagnoses of anorexia nervosa and bulimia nervosa (exact definitions available on request) based on the precise diagnostic criteria specified in DSM-IV and employing a three-month time frame. Those eating disorders that did not meet the EDE-based operational definitions of anorexia nervosa or bulimia nervosa were classed as cases of eating disorder NOS. In addition, diagnoses of “binge eating disorder” were made based on the research criteria specified in DSM-IV using their six-month time frame. Binge eating disorder is a provisional new eating disorder diagnosis that is at present subsumed under the rubric of eating disorder NOS.

### Statistical methods

The data are reported as mean (SD) for continuous data and *N* (%) for categorical data. Patients with the two most common eating disorders, eating disorder NOS and bulimia nervosa, were compared using independent sample *t*-tests or Mann–Whitney tests for continuous data (for normally or non-normally distributed data respectively) and chi-squared tests for categorical data. A cluster analysis based on the four continuous EDE subscale scores (Restraint, Eating Concern, Shape Concern, Weight Concern) was performed with the SPSS 13.0 TwoStep Cluster Analysis procedure to determine automatically the optimal number of clusters within the data. Statistical significance was taken at the 5% level (*p*<0.05) throughout, with 95% confidence intervals (CIs) used to express the uncertainty around the estimates.

## Results

### Sample

The sample comprised 170 out of 175 potential participants: five did not re-attend after their initial assessment. One hundred and five of the participants were from Oxford and 65 from Leicester. Their EDE-based operational DSM-IV diagnoses were as follows: anorexia nervosa—eight participants (4.7%); bulimia nervosa—60 participants (35.3%); eating disorder NOS—102 participants (60.0%). Seven participants (4.1%) fulfilled the DSM-IV research diagnostic criteria for binge eating disorder.

### Aim 1—Clinical characteristics of the eating disorder NOS cases

Table 1 shows the characteristics of the full sample and those of the three DSM-IV diagnostic subgroups. The great majority of the eating disorder NOS cases were female, single and in their twenties, as were those with anorexia nervosa or bulimia nervosa. Their eating disorder was longstanding, the mean duration being 8.2 years (SD=7.2), and generally its course was unremitting. Almost a quarter (22.5%) had a history of anorexia nervosa and 38.2% a history of bulimia nervosa. The EDE ratings of the eating disorder NOS cases indicated that they shared the eating habits and attitudes to shape and weight that characterise anorexia nervosa and bulimia nervosa. They had markedly elevated dietary restraint and concerns about shape, weight and eating: in three-quarters (73.5%) the overall severity of the eating disorder (global EDE score) was more than two SDs above the community norm for young women their age (Beglin, 1990). Almost half (46.1%) reported recurrent objective bulimic episodes and a similar proportion (49.0%) reported self-induced vomiting. A quarter (24.5%) misused laxatives. Forty-two (41.2%) engaged in regular “purging” (i.e., inducing vomiting or misusing laxatives at least twice a week) but did not meet diagnostic criteria for bulimia nervosa. Of these, three-quarters ( $\frac{31}{42}$, 73.8%) reported subjective bulimic episodes and in 19 (45%) these occurred at least twice weekly and so resembled cases of bulimia nervosa albeit with binges that were not large (i.e., subjective bulimic episodes in EDE terms). This state has been referred to as “subjective bulimia nervosa” or “purging disorder” (Keel, Haedt & Edle, 2005; Keel, Mayer & Harnden-Fischer, 2001).

The eating disorder NOS cases had a high level of general psychiatric symptoms, the BSI global severity index being higher in the Leicester patients than the Oxford ones (mean=1.74, SD=0.82 vs mean=1.48, sd=0.76, respectively; Mann–Whitney *z*=2.12, *p*=0.034). The mean BMI (weight in kg/(height in m)^2^) of the eating disorder NOS cases was unremarkable at 22.3 (SD=4.4), the Oxford patients having a lower BMI than the Leicester ones (mean=21.9, SD=3.8 vs mean=23.6, SD=5.2, respectively; *t*=2.33, df=160, *p*=0.021). Eight (7.8%) of the eating disorder NOS cases had a BMI of 17.5 or below, and eight (7.8%) had a BMI of 30 or more.

Comparison of the eating disorder NOS cases with a history of anorexia nervosa with the remainder revealed just one statistically significant difference: those with a history of anorexia nervosa had a lower current BMI (mean=20.2, SD=3.8 vs mean=22.9, SD=4.3; *t*=2.66, df=94, *p*=0.009). Similarly, comparison of those with a history of anorexia nervosa or bulimia nervosa with the remainder revealed one significant difference: those with a positive history had a higher level of general psychiatric symptoms, as measured using the BSI global severity index (mean=1.81, SD=0.76 vs mean=1.24, SD=0.74; Mann–Whitney *z*=3.44, *p*=0.001).

### Aim 2—Comparison of the cases of eating disorder NOS with cases of bulimia nervosa

The cases of eating disorder NOS were compared with those with bulimia nervosa with respect to their demographic features, the full range of their psychopathology and the duration of their eating disorder. Certain differences in their behaviour were present by definition as they were a consequence of how the two disorders are defined.

The two groups were remarkably similar and this close similarity was evident whether or not the analyses were adjusted for site. There were just three statistically significant differences (*p*<0.05): the EDE shape and weight concern subscale ratings and current alcohol intake were higher in the bulimia nervosa patients. With regard to the observed difference in shape and weight concern, the 95% CIs indicate that the likely true differences between the two diagnoses are modest in clinical terms (at most less than one rating point out of the seven on the EDE).

**Fig. 1:**
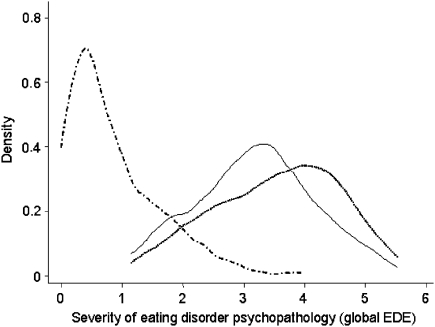
Severity of eating disorder features (global EDE score) in cases of eating disorder NOS (——), cases of bulimia nervosa (- - - -) and in a normative sample of young adult women (·–·–·) (from Beglin, 1990).

Fig. 1 shows the distribution of the global EDE scores of the bulimia nervosa and eating disorder NOS cases, together with that of a representative sample of young adult women from across Oxfordshire (Beglin, 1990). The similarity of the two patient groups is striking as is their difference from the normative sample. Further evidence of their similarity came from a cluster analysis based on the four EDE subscale scores and employing the whole sample. Two clusters emerged (*n*=134 and 36), the smaller having lower global EDE scores. Each cluster contained similar proportions of patients with bulimia nervosa (36% and 33%) and eating disorder NOS (59% and 64%) (*χ*^2^=0.53, df=2, *p*=0.77).

### Aim 3—Impact on the relative prevalence of eating disorder NOS of relaxing the diagnostic criteria for anorexia nervosa and bulimia nervosa

To determine whether the high relative prevalence of eating disorder NOS (60.0%) might be due in part to the presence within eating disorder NOS of cases closely resembling anorexia nervosa and bulimia nervosa, the diagnostic criteria for these two disorders were systematically relaxed along lines advocated in the literature (Cachelin & Maher, 1998; Garfinkel, Kennedy & Kaplan, 1995; Garfinkel et al. 1996; Lee & Kwok, 2005; Niego, Pratt & Agras, 1997; Palmer, 1993, 2003; Pratt, Niego & Agras, 1998; Rieger, Touyz, Swain & Beumont, 2001; Watson & Andersen, 2003). The following changes were made one-by-one and then in combination:

• Anorexia nervosa: 1. Removal of the amenorrhoea criterion. 2. Raising of the BMI threshold to BMI ⩽18.0. 3. Raising it still further to BMI ⩽18.5. 4. Adding those patients whose self-evaluation was judged largely or exclusively on the basis of their ability to control their eating *per se* (rather than only including patients who over-evaluated controlling their eating in order to influence their shape or weight).
• Bulimia nervosa: 1. Reduction of the minimum average frequency of binge eating and purging from two episodes per week to one episode per week. 2. Expansion of the definition of a binge to include episodes of uncontrolled eating that did not involve the consumption of a large amount of food (i.e., the inclusion of subjective bulimic episodes).

Table 2 shows the impact of each of these changes on the relative prevalence of eating disorder NOS, anorexia nervosa and bulimia nervosa. It can be seen that none of the changes in isolation had much effect. Similarly, but with one exception, when all the changes were applied in combination half the sample still retained the diagnosis eating disorder NOS. The exception was when the expanded definition of a binge was applied (i.e., when a binge was defined as any episode of uncontrolled overeating regardless of the amount eaten) together with all the other changes. This resulted in a more notable drop in the relative prevalence of eating disorder NOS and a corresponding increase in the prevalence of bulimia nervosa; nevertheless, over a third (36.5%) of the sample still retained the diagnosis eating disorder NOS. Removal from the existing disorder NOS group of all the subthreshold cases of anorexia nervosa and bulimia nervosa had little effect on the severity of its psychopathology: the remaining cases were still very similar to the core cases of bulimia nervosa.

## Discussion

The first two aims of this study were to describe the clinical characteristics of patients with the DSM-IV diagnosis eating disorder NOS and establish its severity with reference to bulimia nervosa. This required recruiting a large and representative patient sample and assessing it using standardised measures of eating disorder-specific and general psychopathology. This was done. The diagnostic composition of the sample was as expected with over half the patients receiving the diagnosis eating disorder NOS, a third meeting diagnostic criteria for bulimia nervosa, and the remainder having anorexia nervosa. These figures are similar to those from three other studies of well-diagnosed outpatient samples of adults with an eating disorder (Martin et al., 2000; Ricca et al., 2001; Turner & Bryant-Waugh, 2004).

The patients with eating disorder NOS had longstanding eating problems, the mean duration being over eight years. Their EDE ratings showed that they displayed the psychopathology characteristic of anorexia nervosa and bulimia nervosa, and it was comparable in severity to that seen in bulimia nervosa. This remained the case even after the subthreshold cases of anorexia nervosa and bulimia nervosa had been removed. The eating disorder NOS patients resembled those with bulimia nervosa in many other ways: for example, in age, gender, ethnicity, marital status and occupation. In addition, they had similarly raised levels of comorbid general psychiatric symptoms, although their alcohol intake was not as high. Almost a quarter had a history of anorexia nervosa and over third had a history of bulimia nervosa illustrating the cross-diagnostic temporal movement that is common among people with eating disorders (Fairburn & Harrison, 2003; Milos, Spindler, Schnyder, & Fairburn, 2005).

The third aim of the study was to determine whether the high relative prevalence of eating disorder NOS might be due in part to the presence within the diagnosis of cases closely resembling anorexia nervosa or bulimia nervosa, cases that might be better re-diagnosed as such (Fairburn & Bohn, 2005). The availability of EDE ratings on the full sample allowed us to examine this possibility by systematically relaxing the diagnostic criteria for anorexia nervosa and bulimia nervosa along lines already proposed in the literature while still preserving the core clinical concepts (e.g., that patients with anorexia nervosa be actively maintaining an unequivocally low weight, and that patients with bulimia nervosa experience repeated episodes of binge eating). It emerged that, with one exception, none of the adjustments either in isolation or in combination had much impact on the relative prevalence of eating disorder NOS which remained at 50% or more. The exception involved the expansion of the concept of a binge to include any episode of eating associated with a sense of loss of control irrespective of the amount eaten. When this change was made, together with all the other changes, it resulted in an increase in the relative prevalence of bulimia nervosa and corresponding drop in the prevalence of eating disorder NOS but even so over a third of the patients retained the diagnosis eating disorder NOS. Only one other study has systematically investigated the impact of adjusting the diagnostic criteria for anorexia nervosa and bulimia nervosa (Thaw, Williamson, & Martin, 2001) and it used a convenience sample that was likely to have been atypical in composition. Nevertheless similar findings emerged.

It should also be noted that the high relative prevalence of eating disorder NOS was not due to the presence of cases of binge eating disorder as just seven patients met its diagnostic criteria—this figure rose to nine if the minimum average frequency of binge eating was reduced to one day per week. This low prevalence of binge eating disorder is consistent with findings from other samples; for example, two of the three patient samples referred to above reported prevalence figures for binge eating disorder and in both cases it was less than 10% (Martin et al., 2000; Ricca et al., 2001).

This study had certain strengths. First, the two-site catchment area sampling frame meant that the patients were likely to have been representative of many other outpatient samples of adults with eating disorders. Second, the cases were well characterised. Leading measures of psychopathology were employed, and the use of operational EDE-based diagnostic criteria enabled diagnostic thresholds to be adjusted in a systematic and replicable way. Third, although there was multiple statistical testing, we chose not to adjust our significance level when comparing the eating disorder NOS cases with those with bulimia nervosa. This favoured the identification of false positives rather than false negatives (i.e., raising Type I rather than Type II error). This was a conservative strategy since it made more likely the detection of statistically significant differences between the groups. If we had reduced our significance level to the 1% level, none of the observed differences would have been statistically significant.

In terms of limitations, the sample did not include patients at the two far ends of the weight spectrum (i.e., those with a BMI below 16.0 or of 40.0 or above). Patients with a BMI below 16 would have been likely to fulfil diagnostic criteria for anorexia nervosa and those with a BMI of 40 or more would have been likely to be cases of eating disorder NOS as patients with bulimia nervosa are rarely so heavy. Data from the entire Leicester eating disorder service for the three-year period 2003–2005 indicate that both weight groups are uncommon (8% $\left(\frac{39}{490}\right)$ and 5% $\left(\frac{23}{490}\right)$ respectively, of the overall referrals). If they had been included in the present sample, and given their likely diagnoses, the diagnostic distribution would have been 11% anorexia nervosa, 31% bulimia nervosa and 58% eating disorder NOS, figures that differ little from those of the actual study sample. The exclusion of these two small subgroups of patients will have had very little effect on the study's findings since these concern the characteristics of the patients with eating disorder NOS, almost all of whom were included. One other point about the sample should be noted. The study was of adults: its findings cannot necessarily be generalised to adolescents. This said, data on such patients suggest that eating disorder NOS is common among them too (Chamay-Weber, Narring, & Michaud, 2005; Nicholls, Chater, & Lask, 2000).

Four main conclusions may be drawn from this study. First, it is confirmed that eating disorder NOS is the most common eating disorder diagnosis encountered in adult outpatient settings. Second, the psychopathology of eating disorder NOS closely resembles that of anorexia nervosa and bulimia nervosa. Third, across a wide range of clinical variables, eating disorder NOS is comparable in severity to bulimia nervosa. Fourth, the high relative prevalence of eating disorder NOS is not attributable to the existence within the diagnosis of cases closely resembling anorexia nervosa or bulimia nervosa, nor is it due to the presence of cases of binge eating disorder.

The implications of the findings are important, especially with regard to the classification of eating disorders. If NOS diagnoses are intended to be truly “residual”, and by implication few in number, then the clinical state (or states) currently embraced by the diagnosis eating disorder NOS ought to be reclassified as one or more specific forms of eating disorder, especially since it (or they) are as severe as the established eating disorder bulimia nervosa. From these findings it seems reasonable that a small proportion of these cases be re-diagnosed as cases of anorexia nervosa or bulimia nervosa, or binge eating disorder if it becomes a recognised diagnostic entity. However, the great majority differ in their precise clinical presentation whilst still having the psychopathology that characterises anorexia nervosa and bulimia nervosa. How their clinical picture should be classified is a matter of debate (Beumont, Kopec-Schrader, Talbot & Touyz, 1993; Fairburn & Bohn, 2005; Murphy, Perkins, & Schmidt, 2005; Palmer & Norring, 2005). Our general clinical experience and our knowledge of the cases that comprise the present sample suggest that most might be best characterised as “mixed” because the clinical features of anorexia nervosa and bulimia nervosa are present but combined in subtly different ways to those seen in the two currently specified syndromes. Indeed, it has been suggested that such cases could be designated as belonging to a new diagnostic category, perhaps termed “mixed eating disorder” (Fairburn & Bohn, 2005). Alternatively, subcategories of eating disorder NOS could be sought, although we would argue against the delineation of new subgroups unless there is a strong case for doing so. It has been stated that “In the last resort all diagnostic concepts stand or fall by the strength of the prognostic and therapeutic implications they embody” (Kendell, 1975). If this view is accepted, then there is at present no case for subdividing eating disorder NOS since almost nothing is known about the course of these cases or their response to treatment.

The findings also have important practical implications. They highlight the need for studies that recruit broader samples than at present. Research needs to address the whole range of eating disorders seen in clinical practice, not just anorexia nervosa and bulimia nervosa. The recruitment of complete transdiagnostic samples (i.e., those including patients with anorexia nervosa, bulimia nervosa and all forms of eating disorder NOS) would be of special value it would permit the entire scheme for classifying eating disorders to be examined afresh. As noted elsewhere (Fairburn & Bohn, 2005), the collection of good transdiagnostic data, particularly cross-diagnostic information on course and response to treatment, is needed if clinically informative subdivisions are to be identified.

From the clinical point of view there is also a pressing need for studies of the treatment of eating disorder NOS as these patients have been neglected to date. Existing treatments for anorexia nervosa and bulimia nervosa might benefit them, given that they share the same distinctive psychopathology (Fairburn, Cooper, & Shafran, 2003), but this needs to be shown to be the case.

## Figures and Tables

**Table 1:** The clinical characteristics of the eating disorder sample and the three diagnostic subgroups

	Full eating disorder sample (*N*=170)	Eating disorder NOS (*N*=102)	Bulimia nervosa (*N*=60)	Anorexia nervosa (*N*=8)	Comparison of eating disorder NOS and bulimia nervosa	
					Difference^a^ (95% CI)	Test statistic and *p*-value
Age, years, mean (SD)	25.8 (6.8)	26.1 (7.2)	25.5 (6.7)	24.1 (3.4)	0.62 (−1.62, 2.87)	*t*=0.55, df=160, *p*=0.59
Gender, *n* (%) female	162 (95.3)	99 (97.1)	55 (91.7)	8 (100)	5.4% (−1.7, 15.3)	*χ*^2^=1.33, df=1, *p*=0.25
Ethnicity, *n* (%)						
White	152 (89.4)	90 (88.2)	54 (90.0)	8 (100)	White vs other	*χ*^2^ (white vs other)
Asian	12 (7.1)	8 (7.8)	4 (6.7)	0	−1.8% (−11.1, 9.5)	=0.007, df=1, *p*=0.93
African British	0	0	0	0		
Mixed	6 (3.5)	4 (3.9)	2 (3.3)	0		
Marital status, *n* (%)						
Single, never married	129 (75.9)	75 (73.5)	47 (78.3)	7 (87.5)	Single vs other	*χ*^2^ (single vs other)
Married or living as such	38 (22.4)	26 (25.5)	11 (18.3)	1 (12.5)	−4.8% (−17.4, 9.4)	0.246, df=1, *p*=0.62
Separated or divorced	3 (1.8)	1 (1.0)	2 (3.3)	0		
Occupation, *n* (%)						
Higher	30 (17.6)	17 (16.7)	11 (18.3)	2 (25.0)	Student vs other	*χ*^2^ (student vs other)
Intermediate	25 (14.7)	15 (14.7)	9 (15.0)	1 (12.5)	0.78% (−14.8, 16.0)	0.00, df=1, *p*=1.00
Lower	22 (12.9)	13 (12.7)	8 (13.3)	1 (12.5)		
Unclassifiable	18 (10.6)	12 (11.8)	6 (10.0)	0		
Full-time students	75 (44.1)	45 (44.1)	26 (43.3)	4 (50.0)		
Eating disorder psychopathology, mean (SD)						
Overall severity (global EDE^b^)	3.35 (1.0)	3.20 (1.0)	3.49 (1.1)	4.15 (1.0)	−0.29 (−0.61, 0.039)	*z*=1.85, *p*=0.065
Above 1SD of community norm^c^, n (%)	159 (93.5)	94 (92.2)	57 (95.0)	8 (100)	−2.8% (−10.5, 6.7)	*χ*^2^=0.14, df=1, *p*=0.71
Above 2SD of community norm^c^, n (%)	129 (75.9)	75 (73.5)	47 (78.3)	7 (87.5)	−4.8% (−17.4, 9.4)	*χ*^2^=0.25, df=1, *p*=0.62
Dietary restraint (EDE subscale^b^)	3.50 (1.4)	3.43 (1.5)	3.50 (1.3)	4.48 (1.1)	−0.07 (−0.53, 0.39)	*z*=0.17, *p*=0.87
Eating concern (EDE subscale^b^)	2.42 (1.3)	2.23 (1.2)	2.58 (1.4)	3.65 (1.3)	−0.35 (−0.77, 0.065)	*z*=1.49, *p*=0.14
Shape concern (EDE subscale^b^)	3.84 (1.2)	3.64 (1.2)	4.06 (1.1)	4.58 (1.0)	−0.42 (−0.80, 0.036)	*z*=2.21, *p*=0.027
Weight concern (EDE subscale^b^)	3.63 (1.3)	3.51 (1.2)	3.81 (1.4)	3.90 (1.5)	−0.30 (−0.71, 0.11)	*z*=1.99, *p*=0.047
Eating disorder behaviour						
Objective bulimic episodes, *n* (%) present	108 (63.5)	47 (46.1)	60 (100)	1 (12.5)	−53.9% (−63.3, −42.6)	*χ*^2^=46.6, df=1, *p*<0.001
If present, episodes/28 days (median)	14.5	6.0	22.0	29.0		*z*=6.54, *p*<0.001
Bulimic episodes of any size, *n* (%) present	148 (87.1)	80 (78.4)	60 (100)	8 (100)	−21.6% (−30.5, −12.4)	*χ*^2^=13.19, df=1, *p*<0.001
If present, episodes/28 days (median)	22.5	12.0	30.0	14.5		*z*=5.91, *p*<0.001
Self-induced vomiting, *n* (%) present	104 (61.2)	50 (49.0)	52 (86.7)	2 (25.0)	−37.7% (−49.1, −23.2)	*χ*^2^=21.37, df=1, *p*<0.001
If present, episodes/28 days (median)	20.5	8.0	30.0	37.5		*z*=5.19, *p*<0.001
Laxative misuse, *n* (%) present	43 (25.3)	25 (24.5)	14 (23.3)	4 (50.0)	1.2% (−13.0, 14.0)	*χ*^2^=0.00, df=1, *p*=1.00
If present, episodes/28 days (median)	10.0	7.0	20.5	11.0		*z*=1.92, *p*=0.054
Body mass index, mean (SD)	22.3 (4.4)	22.3 (4.3)	23.0 (4.3)	16.7 (0.6)	−0.82 (−2.26, 0.62)	*t*=1.12, df=160, *p*=0.26
Duration of eating disorder, years, mean (SD)	8.3 (7.0)	8.2 (7.2)	9.0 (6.8)	3.8 (2.8)	−0.87 (−3.13, 1.40)	*t*=0.76, df=160, *p*=0.45
Lowest adult body mass index, mean (SD)	18.4 (2.9)	18.3 (2.9)	18.9 (2.9)	16.3 (0.9)	−0.60 (−1.57, 0.36)	*t*=1.24, df=149, *p*=0.22
Highest adult body mass index, mean (SD)	25.9 (4.9)	25.9 (4.7)	26.4 (5.4)	21.8 (1.8)	−0.49 (−2.09, 1.10)	*t*=0.61, df=158, *p*=0.54
General psychiatric features, BSI^d^, mean (SD)	1.61 (0.8)	1.55 (0.8)	1.62 (0.8)	2.25 (0.8)	−0.08 (−0.33, 0.18)	*t*=0.58, df=156, *p*=0.57
Self-esteem, RSE^e^, mean (SD)	21.1 (5.5)	21.3 (5.0)	21.1 (6.0)	17.0 (5.4)	0.21 (−1.72, 2.14)	*t*=0.22, df=127, *p*=0.83
Social adjustment, SAS^f^, mean (SD)	1.57 (0.5)	1.54 (0.4)	1.59 (0.5)	1.87 (0.4)	−0.055 (−0.21, 0.10)	*t*=0.68, df=128, *p*=0.50
Alcohol intake, units/week, mean (SD)	10.9 (15.1)	9.6 (15.5)	14.1 (14.7)	3.4 (2.5)	−4.43 (−9.32, 0.46)	*z*=2.54, *p*=0.011
Alcohol intake, *n* (%) with mean weekly intake above UK recommended level	44 (25.9)	24 (23.5)	20 (33.3)	0	−9.8% (−24.3, 4.17)	*χ*^2^=1.37, df=1, *p*=0.24
Drug misuse, current, *n* (%)	3 (1.8)	3 (2.9)	0	0	−2.9% (−3.4, 8.3)	*χ*^2^=0.54, df=1, *p*=0.46
Self-harm, current, *n* (%)	25 (14.7)	12 (11.8)	11 (18.3)	2 (25.0)	−6.6% (−19.2, 4.36)	*χ*^2^=0.85, df=1, *p*=0.36

a Eating disorder NOS score minus bulimia nervosa score.

b Eating Disorder Examination (Fairburn & Cooper, 1993).

c Community EDE norm for young adult women (Beglin, 1990).

d Brief Symptom Inventory (Derogatis & Spencer, 1982).

e Rosenberg Self-esteem Scale (Rosenberg, 1965).

f Social Adjustment Scale—UK version (Cooper et al., 1982).

**Table 2:** Impact on the relative prevalence of the three DSM-IV eating disorders of relaxing the diagnostic criteria for anorexia nervosa and bulimia nervosa

	Anorexia nervosa *N* (%)	Bulimia nervosa *N* (%)	Eating disorder NOS *N* (%)
1. *Current DSM-IV diagnostic criteria*	8 (4.7)	60 (35.3)	102 (60.0)
2. *Adjustments to the anorexia nervosa criteria*:			
a. Removal of the amenorrhea criterion	12 (7.1)	58 (34.1)	100 (58.8)
b. Raising of the BMI threshold to <18.0 (kg/m^2^)	8 (4.7)	60 (35.3)	102 (60.0)
c. Raising of the BMI threshold to <18.5 (kg/m^2^)	12 (7.1)	58 (34.1)	100 (58.8)
d. Addition of those who overvalue control over eating *per se*	8 (4.7)	60 (35.3)	102 (60.0)
e. Adjustments a, c and d combined	21 (12.4)	56 (32.9)	93 (54.7)
3. *Adjustments to the bulimia nervosa criteria*:			
a. Reduction of the minimum average frequency of binge eating and purging to at least once per week	8 (4.7)	68 (40.0)	94 (55.3)
b. Expansion of the definition of binge eating to include subjective bulimic episodes	8 (4.7)	80 (47.1)	82 (48.2)
4. *Adjustments to the anorexia nervosa and bulimia nervosa criteria combined*:			
a. All, excluding adjustment 3b	21 (12.4)	64 (37.6)	85 (50.0)
b. All, including adjustment 3b	21 (12.4)	87 (51.2)	62 (36.5)
